# Semiquantitative assessment of subchondral bone marrow edema-like lesions and subchondral cysts of the knee at 3T MRI: A comparison between intermediate-weighted fat-suppressed spin echo and Dual Echo Steady State sequences

**DOI:** 10.1186/1471-2474-12-198

**Published:** 2011-09-09

**Authors:** Daichi Hayashi, Ali Guermazi, C Kent Kwoh, Michael J Hannon, Carolyn Moore, John M Jakicic, Stephanie M Green, Frank W Roemer

**Affiliations:** 1 Quantitative Imaging Center, Department of Radiology, Boston University School of Medicine, Boston, MA 02118, USA; 2 Department of Radiology, Klinikum Augsburg, Augsburg, Germany; 3 Division of Rheumatology and Clinical Immunology, University of Pittsburgh School of Medicine, Pittsburgh, PA 15261, USA; 4 Pittsburgh VA Healthcare System, Pittsburgh, PA 15240, USA; 5 Texas Woman's University, Houston, TX 77030, USA; 6 Department of Health and Physical Activity, University of Pittsburgh, Pittsburgh, PA 15260, USA

**Keywords:** bone marrow lesion, cyst, MRI, knee, osteoarthritis

## Abstract

**Background:**

Choice of appropriate MR pulse sequence is important for any research studies using imaging-derived data. The aim of this study was to compare semiquantitative assessment of subchondral bone marrow edema-like lesions and subchondral cysts using intermediate-weighted (IW) fat-suppressed (fs) spin echo and Dual Echo Steady State (DESS) sequences on 3 T MRI.

**Methods:**

Included were 201 subjects aged 35-65 with frequent knee pain. 3T MRI was performed with the same sequence protocol as in the Osteoarthritis Initiative (OAI). In a primary reading subchondral bone marrow edema-like lesions were assessed according to the WORMS system. Two hundred subregions with such lesions were randomly chosen. The extent of subchondral bone marrow edema-like lesions was re-evaluated separately using sagittal IW fs and DESS sequences according to WORMS. Lesion size and confidence of the differentiation between subchondral bone marrow edema-like lesions and subchondral cysts located within or adjacent to them was rated from 0 to 3. Wilcoxon signed-rank tests and chi-square statistics were used to examine differences between the two sequences.

**Results:**

Of 200 subchondral bone marrow edema-like lesions detected by IW fs sequence, 93 lesions (46.5%) were not depicted by the DESS sequence. The IW fs sequence depicted subchondral bone marrow edema-like lesions to a larger extent than DESS (p < 0.0001), and the opposite was true for subchondral cysts. Confidence scores for differentiation of the two types of lesions were not significantly different between the two sequences.

**Conclusions:**

In direct comparison the IW fs sequence depicts more subchondral bone marrow edema-like lesions and better demonstrate the extent of their maximum size. The DESS sequence helps in the differentiation of subchondral bone marrow edema-like lesions and subchondral cysts. The IW fs sequence should be used for determination of lesion extent whenever the size of subchondral bone marrow edema-like lesions is the focus of attention.

## Background

Subchondral bone marrow edema-like lesions (BML) are defined as non-cystic areas of ill-delineated hyperintensity on fluid-sensitive fast spin echo (FSE) fat suppressed (fs) pulse sequences and of hypointensity on T1-weighted (T1W) spin echo (SE) images [1]. They are one of the features of osteoarthritis (OA) detected on MRI and are observed regularly in conjunction with structural alterations of adjacent cartilage. Higher prevalence and greater volume of concomitant BMLs has been reported to associated be with higher grades of cartilage loss [2].

As OA progresses, an increase in BML volume is seen in the subchondral bone in many patients, and this is positively correlated with an increase in cartilage loss in the same region [2,3]. Subchondral cysts may be present within or adjacent to a BML [4]. They are identified as foci of markedly increased signal in the subchondral bone with well delineated margins and no evidence of internal marrow tissue or trabecular bone. Semiquantitative assessment of subchondral BMLs and cysts is commonly performed on FSE sequences such as T2-weighted (T2W), intermediate-weighted (IW) or proton density-weighted (PDW) fs sequences [5] or short-tau inversion recovery (STIR) sequence [6]. However, BMLs have also been assessed on gradient recalled echo (GRE)-type sequences such as Fast Low Angle Shot (FLASH) or Spoiled Gradient Recalled (SPGR) [7] that are commonly used for quantitative assessment of cartilage volume and thickness due to their high contrast of cartilage to subchondral bone [8]. There is an ongoing discussion regarding the choice of MR pulse sequences that would optimize BML assessment [9]. In light of this debate, a head-to-head comparison of FSE and GRE sequences for semiquantitative assessment of BMLs is needed to objectively appreciate potential differences. The Osteoarthritis Initiative (OAI) MRI protocol with sagittal IW fs and DESS (Dual Echo Steady State, which is a T2-weighted gradient echo sequence) sequences acquired at 3 T MRI allows such a comparison.

The aim of our study was a comparison of semiquantitative assessment of subchondral BMLs and subchondral cysts using the DESS and IW fs sequences at 3 T MRI. Due to lack of a definitive reference standard, this study is primarily aimed at demonstrating how visualization of subchondral BMLs and cysts differs by sequence and at highlighting the strengths and weaknesses of each sequence for assessment of those lesions.

## Methods

### Study subjects

Subjects included in the present study were participants in the Joints On Glucosamine (JOG) cohort. The JOG study is a 6-month double-blind randomized controlled trial to examine the efficacy of oral glucosamine supplementation. Two hundred and one participants, aged 35 to 65, with mild to moderate chronic, frequent knee pain (Western Ontario and McMaster Universities (WOMAC) score ≥ 25 [10]) were recruited at the University of Pittsburgh, Pittsburgh, PA. Subjects were excluded from JOG if they screened positive for rheumatoid arthritis; had ankylosing spondylitis, psoriatic arthritis, chronic reactive arthritis, or renal insufficiency that required hemo- or peritoneal dialysis; were taking bisphosphonates or dietary supplements for knee pain in the 6 months prior to study entry; had a history of cancer (except for non-melanoma skin cancer); had or planned to have bilateral knee replacement surgery; or were unable to walk without assistance. No BMLs of non-degenerative origin (e.g. trauma) were found in this study.

The baseline and follow-up MRI examinations of both knees, when possible, of the 177 subjects who completed the study were examined. Due to previous total knee arthroplasty or the presence of radiographic end-stage OA, eight participants had only one knee scanned, leaving 346 knees that were included in the analyses. Although the JOG Study itself was a longitudinal study, the present study only involves a cross-sectional analysis based on the MRI examinations taken at the baseline.

Institutional Review Board approval and all participants' written informed consent were obtained for this study.

### MRI Acquisition

3 T MRI (Siemens Trio, Erlangen, Germany) was acquired on the same MRI scanner that is used at the Pittsburgh site of the OAI. The identical pulse sequence protocol used for the OAI was applied in the JOG study, excluding the FLASH sequence and the Multi-Echo Spin Echo T2 mapping sequence. Details of the full OAI pulse sequence protocol and the sequence parameters have been published [11]. The protocol included a sagittal 3D DESS sequence with water excitation (WE) (slice thickness = 0.7 mm, interslice gap = 0 mm, repetition time (TR) = 16.3 ms, echo time (TE) = 4.7 ms, flip angle = 25°, field of view (FOV) = 140 mm × 140 mm, matrix = 384 × 307 pixels, echo train length = 1, number of slices = 35, bandwidth = 185 Hz/pixel, number of excitations = 1, anterior/posterior phase encoding axis, acquisition time = 10 minutes 23 seconds), and the sagittal intermediate-weighted (IW) fat-suppressed (fs) 2D turbo spin echo (TSE) sequence (slice thickness = 3 mm, interslice gap = 0 mm, TR = 30 ms, TE = 3,200 ms, flip angle = 180°, FOV = 160 mm × 160 mm, matrix = 313 × 448 pixels, echo train length = 5, number of slices = 37, bandwidth = 248 Hz/pixel, number of excitations = 1, anterior/posterior phase encoding axis, acquisition time = 4 minutes 42 seconds).

### MRI Assessment

**Figure 1:**
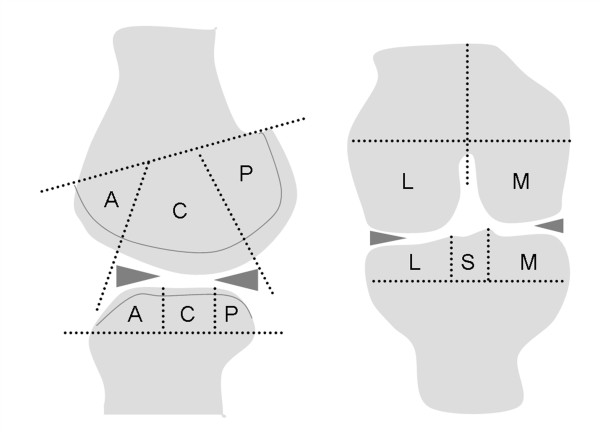
**Regional subdivision of the articular surfaces of the tibiofemoral joint of the knee**. The femur and tibia are divided into lateral (L) and medial (M) regions, with the trochlear groove of the femur considered part of the M region. Region S represents the portion of the tibia beneath the tibial spines. The femoral and tibial surfaces are further subdivided into anterior (A), central (C) and posterior (P) regions. Region A of the femur corresponds to the patellofemoral articulation; region C the weight bearing surface and region P the posterior convexity that articulates only in extreme flexion. Region C of the tibial surface corresponds to the uncovered portion between the anterior and posterior horns of the meniscus centrally and the portion covered by the body of the meniscus peripherally.

**Figure 2:**
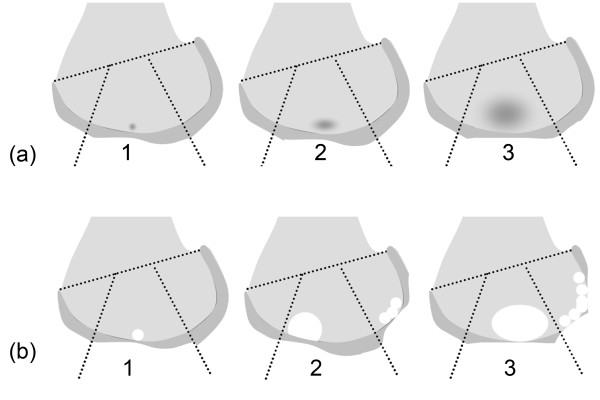
**Schematic illustration of semiquantitative scoring system for subchondral bone marrow edema-like lesions and subchondral cysts using the Whole Organ Magnetic Resonance Imaging Score (WORMS). a:** Scoring of subchondral   bone marrow edema-like lesions. The score is based on the extent of regional marrow involvement by areas of free water   signal with ill-defined margins. **b:** Scoring of subchondral cysts. The score is based on the extent of focal bone loss through   individual cysts (illustrated in the central region) or multiple cysts (illustrated in the posterior region) subchondrally.

One musculoskeletal radiologist (FWR) with 7 years experience of standardized semiquantitative assessment of knee OA, blinded to clinical data, read the baseline MR images of all 346 knees using all 5 available sequences. The MRI evaluation in JOG included the joint features of subchondral BMLs, subchondral cysts, cartilage, meniscus, effusion and synovitis using the Whole Organ Magnetic Resonance Imaging Score (WORMS) method [12]. WORMS is a validated research tool for semiquantitative assessment of knee OA. In WORMS, subchondral BMLs are scored from 0 to 3 based on the extent of subregional involvement (0 = none; 1 = < 25% of the subregion; 2 = 25-50%; 3 = > 50%, Figure 1, 2a). Similarly, subchondral cysts are also scored from 0 to 3 based on the lesion extent in regard to subregional involvement (0 = none; 1 = < 25% of the subregion; 2 = 25-50%; 3 = > 50%, Figure 2b) at baseline. Thus, the WORMS score for subchondral BMLs and cysts is a sum of percentage of subregion for each type of lesion and does not give information on the number of lesions. In the following, we will use the term subchondral BML and subchondral cyst interchangeably for "percentage of the area occupied by BML and cyst within a subregion" as defined in WORMS. Altogether 654 subregions exhibiting subchondral BMLs were observed in 262 knees. Two hundred subregions exhibiting subchondral BMLs from 63 knees of 42 subjects in the primary reading were randomly chosen for the consequent direct sequence comparison. One hundred and ten of these 200 subregions also exhibited subchondral cysts.

In a second consensus reading by two experienced musculoskeletal radiologists (FWR, AG) with 7 and 9 years experience in standardized semiquantitative MR assessment of knee OA, these 200 subregions were re-evaluated using the sagittal 3D DESS and sagittal IW fs TSE sequences only. Readings for both sequences were performed separately with a time interval of 4 weeks to avoid recognition bias. All subregions were re-evaluated for lesion size of subchondral BMLs and cysts. Further, confidence of the differentiation between these two types of lesions was rated on a 0-3 scale (0 = unable to differentiate; 1 = doubtful differentiation; 2 = may be able to differentiate with some confidence; 3 = able to differentiate with definite confidence). Wilcoxon signed-rank tests for paired comparisons of clustered data were used to examine if there were statistically significant differences between the two sequences, and clustering by person was controlled [13]. All analyses were performed using SAS_® _software (Version 9.2 for Windows; SAS Institute, Cary, NC).

## Results

Of the 177 participants who completed the study, mean age at enrollment was 52.3 (SD ± 6.2). There were slightly more men than women (53.7% men) and patients were on average overweight (mean BMI 29.1 ± 4.1). Of the 200 subregions assessed with BML scores > 0 at the primary reading, 88 were found in the left knee and 112 in the right knee; 81 (41%) were found in the medial, 24 (12%) in the lateral tibiofemoral compartments, and 94 (48%) were detected in the patellofemoral compartments (Table 1).

**Table 1:** Distribution of non-cystic bone marrow lesions according to their locations

Location*	Frequency	(Percent)
Tibiofemoral joint	105	(52.5)
Subspinous	27	(13.5)
Lateral femur (central and posterior)	8	(4.0)
Lateral tibia	16	(8.0)
Medial femur (central and posterior)	28	(14.0)
Medial tibia	26	(13.0)
Patellofemoral joint	95	(47.5)
Lateral femur (anterior)	24	(12.0)
Medial femur (anterior)	15	(7.5)
Lateral patella	34	(17.0)
Medial patella	22	(11.0)

* According to the Whole Organ Magnetic Resonance Imaging Score (WORMS), the knee joint is classified into tibiofemoral joint (including subspinous subregion, central and posterior, lateral and medial femoral subregions and lateral and medial tibial subregions) and patellofemoral joint (including anterior lateral and medial femoral subregions, and lateral and medial patellar subregions).

**Figure 3:**
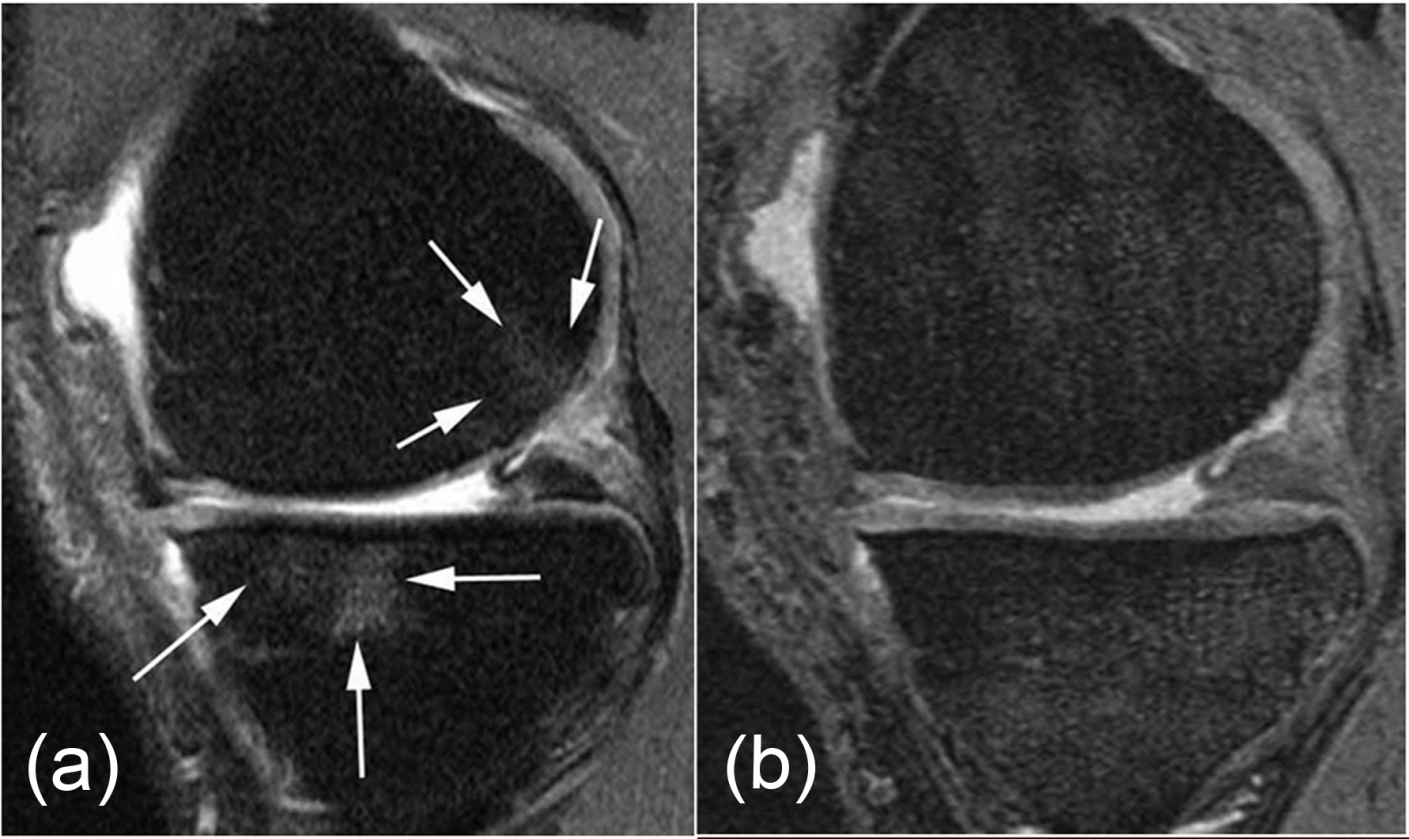
**61-year old woman with medial tibio-femoral knee osteoarthritis**. **a: **Sagittal intermediate-weighted (IW) fat-suppressed image depicts a grade 2 subchondral bone marrow edema-like lesion at the central medial tibial plateau that extends to the anterior subregion (long white arrows show the approximate location of the ill-defined margin of the lesion). In addition, there is a grade 1 subchondral bone marrow edema-like lesion at the posterior medial femur (short white arrows show the approximate location of the ill-defined margin of the lesion). **b: **Sagittal Dual Echo Steady-State (DESS) image shows no bone marrow edema-like lesion in neither the femur nor the tibia.

The IW fs sequence demonstrated size of subchondral BMLs as being larger in 186 (93.0%) subregions when compared to the DESS (Wilcoxon signed-rank test, p < 0.0001) (Table 2). This includes presence of subchondral BMLs on the IW fs sequence and absence on the DESS sequence. These were depicted larger by one grade in 119 (59.5%), by two grades in 52 (26.0%) and by three grades in 15 (7.5%) subregions (Figure 3). For subregions in which subchondral BMLs were depicted in both sequences, the corresponding numbers for one grade and two grade differences (i.e. larger in IW fs sequence) were 64 (59.8%) and 29 (27.1%). Fourteen (7.0%) subregions were scored with the same grade in both sequences. In no case did the IW fs exhibit the lesions as being smaller when compared to the DESS. Ninety three (46.5%) subregions with subchondral BMLs on the IW fs sequence did not exhibit any such lesions on the DESS.

**Table 2:** Comparison of the extent of non-cystic BMLs (n = 200) as semiquantitatively evaluated using intermediate-weighted (IW) fat-suppressed (fs) and Dual Echo Steady-State (DESS) sequences

	IW fs	DESS
Score	Frequency (%)	Frequency (%)

0	0 (0)	93 (46.5)
1	67 (33.5)	100 (50.0)
2	84 (42.0)	7 (3.5)
3	49 (24.5)	0 (0)

*Overall, IW fs sequence demonstrated the lesions to a larger extent than DESS sequence (Wilcoxon signed-rank test controlling for clustering by person, p < 0.0001).

**Figure 4:**
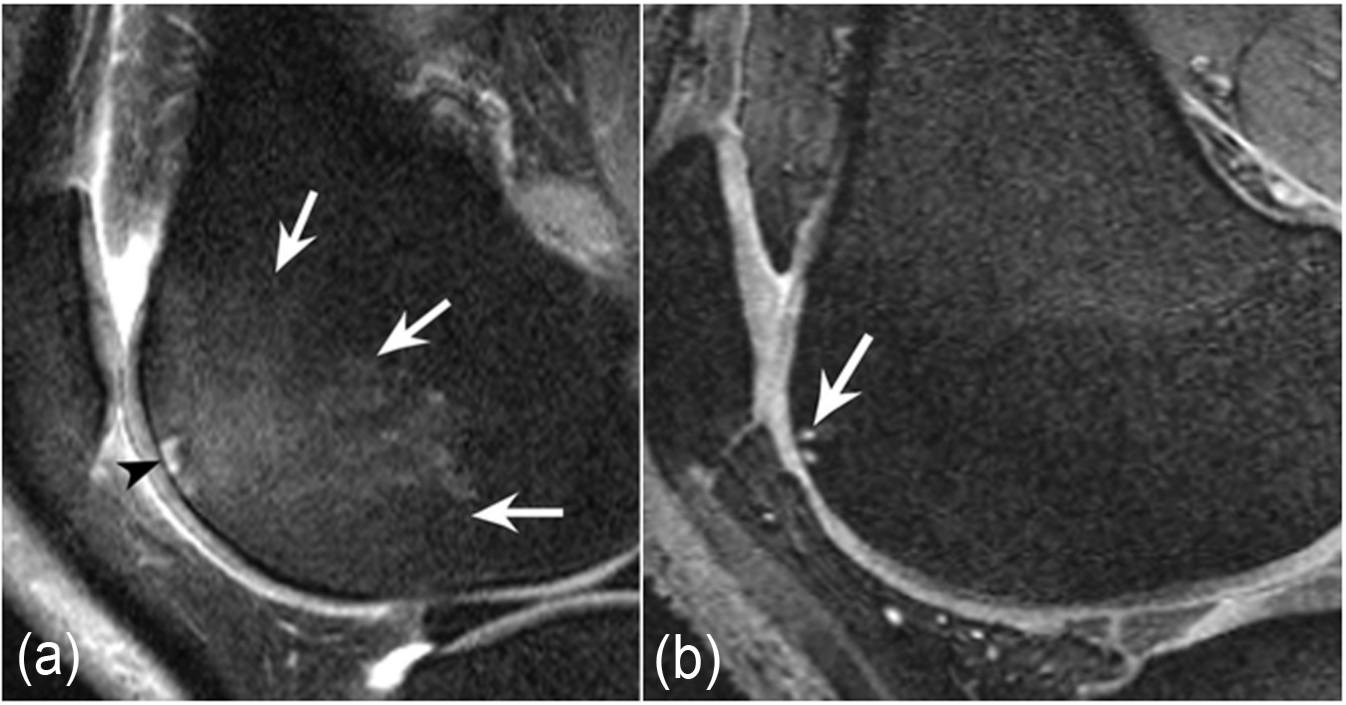
**54-year old woman with knee osteoarthritis**. **a: **Sagittal intermediate-weighted (IW) fat-suppressed (fs) image shows a large (grade 3) subchondral bone marrow edema-like lesion at the lateral femoral trochlea (white arrows). Within this lesion, there is a small subchondral cyst (grade 1) directly adjacent to the subchondral plate (black arrowhead). **b: **Corresponding sagittal Dual Echo Steady-State (DESS) image only shows the small cyst (white arrow). The large bone marrow edema-like lesion is not depicted by the DESS. Consequently, the margin of the cyst is more clearly delineated when compared to IW fs sequence.

In contrast, for subchondral cysts, the DESS sequence demonstrated the lesions to a larger extent than the IW fs sequence in 40 cases (36.4%) (Wilcoxon signed-rank test, p < 0.0001) (Table 3). Seventy six lesions (69.1%) were scored with the same grade in both sequences (Figure 4), and in only one case did the IW fs sequence demonstrate the lesion to a larger extent than the DESS.

**Table 3:** Comparison of the extent of cystic BMLs (n = 113) as semiquantitatively evaluated using intermediate-weighted (IW) fat-suppressed (fs) and Dual Echo Steady-State (DESS) sequences

	IW fs	DESS
**Score**	**Frequency (%)**	**Frequency (%)**

0	17 (15.5)	0
1	73 (66.4)	74 (67.3)
2	13 (11.8)	28 (25.5)
3	7 (6.4)	8 (7.3)

*Overall, DESS sequence demonstrated the lesions to a larger extent than IW fs sequence (Wilcoxon signed-rank test controlling for clustering by person, p < 0.0001).

The distribution of the confidence ratings for the differentiation of subchondral BMLs and cysts varied between the sequences (Table 4), but by the Wilcoxon sign-rank test, the sequences were not different (p = 0.93). The confidence score was higher in the IW fs sequence in 41 lesions (20.5%), higher in DESS sequence in 85 lesions (42.5%), and the scores were the same in 74 (37%) lesions.

**Table 4:** Confidence of differentiation between cystic and non-cystic parts of bone marrow lesions, evaluated using intermediate-weighted (IW) fat-suppressed (fs) and Dual Echo Steady-State (DESS) sequences

	IW fs	DESS
**Score**	**Frequency (%)**	**Frequency (%)**

0	9 (4.5)	38 (19.0)
1	28 (14.0)	0 (0)
2	58 (29.0)	6 (3.0)
3	105 (52.5)	156 (78.0)

*By the Wilcoxon sign-rank test controlling for clustering by person, the difference between the two sequences were not statistically significant (p = 0.9306).

## Discussion

Summarizing our results, we found that subchondral BMLs were more conspicuous and appeared larger when using the sagittal IW fs TSE sequence, compared with the sagittal DESS sequence. Conversely, subchondral cysts appeared larger when using the DESS sequence. Confidence ratings for the differentiation of subchondral BMLs and cysts were not significantly different between the two sequences.

Choice of appropriate pulse sequences is a very important issue in MRI-based OA research. As we have shown, the extent of subchondral BMLs will be underestimated, or lesions might be completely missed by using the DESS sequence. Subchondral BMLs are an important feature of knee OA that is associated with pain [14] and cartilage damage [2]. Multiple publications have utilized MRI-assessment of subchondral BMLs, with most of these applying semiquantitative approaches [2,4,7,15] and others applying quantitative methodology [16-18]. However some of the results presented in these studies should be interpreted carefully, since GRE-type sequences that may not fully depict subchondral BMLs were used [7].

GRE-type sequences, even with robust fat suppression or water excitation, are notoriously insenstitive to bone marrow abnormalities due to trabecular magnetic susceptibility of T2* effects, which may result in underestimation of the size of subchondral BMLs [19,20]. Recent studies have demonstrated that these sequences are also less sensitive in the detection of subchondral BMLs when using FSE sequences as the reference standard [21,22]. These results were summarized and published in a consensus statement by Outcome Measures in Rheumatology Clinical Trials (OMERACT) and Osteoarthritis Research Society International (OARSI) in 2006 [5]. Our results are in line with these previous publications and further strengthen the case that GRE-type sequences are inappropriate for assessment of subchondral BMLs.

It has been shown that contrast-enhanced T1-weighted fs sequences may also be used and may offer equivalent diagnostic performance for subchondral BML evaluation compared to non-enhanced PDw FSE sequences [16], but administration of a contrast agent is not routine for assessment of OA knees, except when synovitis evaluation is the center of attention.

Although we focused on subchondral BMLs that are of degenerative origin only, they can represent a variety of pathologies [23]. Since this study demonstrated their appearance may vary depending on the MRI pulse sequence used, one should be cautious when evaluating BMLs even if they are non-degenerative in origin.

Subchondral cysts are better delineated by the DESS sequence. In this situation, the insensitivity of GRE-type sequences to subchondral BMLs is actually advantageous [5], and the borders between subchondral BMLs and cysts are more clearly delineated than by FSE sequences. The IW fs sequence usually delineates less clearly the sclerotic rim of the cyst when compared to the DESS sequence, and thus a peripheral portion of the cyst might be attributed to be ill-defined on the IW fs sequence. This may be the reason why the cysts appear larger on the DESS sequence.

Thus far, studies have shown no association between the presence of subchondral cysts and pain in subjects with knee OA [24,25], and thus clinical research efforts tend to be more focused on subchondral BMLs, whose association with pain has been clearly demonstrated [14,26]. Ideally, all research protocols should include both a GRE-type sequence and a FSE fs sequence [5], but if practical reasons (e.g. funding issues) limit the number of sequences that can be acquired in a given study, FSE fs sequences should be acquired in preference to GRE-type sequences whenever subchondral BMLs are the focus of study.

Limitations of the present study include a lack of reference standard. Thus, the true size of subchondral BMLs was not assessed by either sequence. One might potentially argue that an IW fs sequence overestimates the extent of the lesion relative to the DESS sequence. Although this cannot be ruled out completely, based upon current knowledge, we believe it is more likely that IW fs depicts the maximum extent of subchondral BMLs [6]. Zanetti et al. showed that the location of 'bone marrow edema-pattern signal alteration' (which is the same as the subchondral BML in our study) on one of the fluid-sensitive non-GRE type sequences (STIR) corresponded to the area of bone marrow edema-like changes as confirmed by histological analysis [6]. No study has confirmed if the extent of subchondral BMLs as seen on GRE-type sequences matched that seen on histological examination. Another limitation that must be noted is that the imaging evaluation occurs at only one time point, and thus we are unable to comment on each sequence's sensitivity to change in a longitudinal study. Lastly, we did not evaluate the state of hyaline cartilage and their appearances in the two types of pulse sequences because it was deemed outside the scope of the present study. However, interested readers are directed to a recently published article which compared semiquantitative assessment of focal cartilage damage using the DESS and IW fs sequences [27]. They demonstrated that the IW fs sequence detected more and larger focal cartilage defects than the DESS, but more intrachondral signal changes were observed with the DESS.

## Conclusions

Summarizing our findings, the maximum extent of subchondral BMLs seems to be depicted on the IW fs sequence when compared directly to the DESS. The DESS sequence helps in the differentiation of subchondral BMLs and cysts, as it depicts cysts as being larger than on the IW fs sequence. Further, the DESS may show only the cysts and not the ill-defined subchondral BMLs. Both sequences appear to be complementary and, based on our results, clear superiority of one sequence over the other could not be demonstrated. However, if the main focus of any study is evaluation of subchondral BMLs, assessment should be performed on FSE fs sequences that depict these lesions to their maximum extent.

## Abbreviations

IW: intermediate-weighted; fs: fat-suppressed; TR: repetition time; TE: echo time; FOV: field of view; DESS: Dual Echo Steady State; FLASH: fast low angle shot; SPGR: spoiled gradient recalled; FSE: fast spin echo; GRE: gradient-recalled echo; STIR: short tau inversion recovery; WORMS: Whole Organ Magnetic Resonance Imaging Score; BML: bone marrow lesion; OAI: Osteoarthritis Initiative; OMERACT: Outcome Measures in Rheumatology Clinical Trials; WOMAC: Western Ontario and McMaster Universities

## Competing interests

Dr. Guermazi has received consultancies, speaking fees, and/or honoraria (less than $10,000 each) from Facet Solutions, Genzyme, Stryker, and (more than $10,000) from Merck Serono, and is the President of Boston Imaging Core Lab (BICL), a company providing image assessment services. He receives research grant funding from General Electric Healthcare. Dr. Roemer is Vice President and shareholder of BICL. Dr. Kwoh receives research grant funding from the Beverage Institute for Health & Wellness, The Coca-Cola Company. None of the other authors have declared any possible conflict of interest.

## Authors' contributions

Guarantors of integrity of the entire study are AG and FWR. Study concepts and design were drawn by DH, AG, CKK, MJH, CM, JMJ, SMG, and FWR. Literature research was performed by DH, AG, and FWR. Clinical studies were performed by AG, CKK, SMG, and FWR. Experimental studies/data analysis were performed by DH, AG, CKK, MJH, and FWR. Statistical analysis was performed by CKK and MJH. All authors contributed to the preparation and editing of this manuscript, and read and approved the final version of the manuscript.

## Funding Sources

The JOG study is funded by a grant from the Coca-Cola Company Beverage Institute for Health & Wellness. The sponsor did not have any role in the study design, analysis and interpretation of data, writing of the manuscript, or the decision to submit the manuscript for publication.

## Pre-publication history

The pre-publication history for this paper can be accessed here:

http://www.biomedcentral.com/1471-2474/12/198/prepub
